# AI Methods Tailored to Influenza, RSV, HIV, and SARS-CoV-2: A Focused Review

**DOI:** 10.3390/pathogens14080748

**Published:** 2025-07-30

**Authors:** Achilleas Livieratos, George C. Kagadis, Charalambos Gogos, Karolina Akinosoglou

**Affiliations:** 1 Independent Researcher, 15238 Athens, Greece; achilleas.livieratos@gmail.com; 2 Department of Medicine, University of Patras, 26504 Rio, Greece; gkagad@gmail.com (G.C.K.); cgogos@med.upatras.gr (C.G.); 3 Metropolitan General Hospital, 15562 Athens, Greece; 4 Department of Internal Medicine and Infectious Diseases, University General Hospital of Patras, 26504 Rio, Greece

**Keywords:** artificial intelligence, influenza, COVID-19, RSV, HIV

## Abstract

Artificial intelligence (AI) techniques—ranging from hybrid mechanistic–machine learning (ML) ensembles to gradient-boosted decision trees, support-vector machines, and deep neural networks—are transforming the management of seasonal influenza, respiratory syncytial virus (RSV), human immunodeficiency virus (HIV), and severe acute respiratory syndrome coronavirus 2 (SARS-CoV-2). Symptom-based triage models using eXtreme Gradient Boosting (XGBoost) and Random Forests, as well as imaging classifiers built on convolutional neural networks (CNNs), have improved diagnostic accuracy across respiratory infections. Transformer-based architectures and social media surveillance pipelines have enabled real-time monitoring of COVID-19. In HIV research, support-vector machines (SVMs), logistic regression, and deep neural network (DNN) frameworks advance viral-protein classification and drug-resistance mapping, accelerating antiviral and vaccine discovery. Despite these successes, persistent challenges remain—data heterogeneity, limited model interpretability, hallucinations in large language models (LLMs), and infrastructure gaps in low-resource settings. We recommend standardized open-access data pipelines and integration of explainable-AI methodologies to ensure safe, equitable deployment of AI-driven interventions in future viral-outbreak responses.

## 1. Introduction

The persistent global burden of viral pathogens such as seasonal influenza, HIV, RSV, and SARS-CoV-2 has driven a surge of interest in harnessing AI to accelerate both diagnostic and therapeutic advances. Although each of these viruses presents distinct epidemiological and immunological challenges, recent years have witnessed a convergence of AI-driven strategies—ranging from computational drug discovery and vaccine design to automated clinical decision support and imaging analysis—that promise to transform our response to these pandemics [1,2,3]. In this review, we apply AI as an umbrella term covering both classical machine-learning techniques and more recent deep-learning or generative-model approaches.

One of the earliest and most impactful applications of AI during the SARS-CoV-2 pandemic was in silico drug discovery and vaccine development [1,2,3,4]. Machine learning (ML) models—especially deep learning architectures—have enabled rapid virtual screening against key viral targets such as the SARS-CoV-2 spike protein and RNA-dependent RNA polymerase [1,2,3,4]. Deep neural networks, including convolutional neural networks (CNNs) for molecular property prediction and attention-based transformers for sequence modeling, have been trained to recognize patterns in large molecular libraries and suggest novel inhibitor scaffolds at unprecedented speed and scale [5,6,7]. Parallel efforts in “reverse vaccinology” have leveraged AI to predict antigenic epitopes directly from viral genomes, automating feature extraction for candidate peptide vaccines [5,6,7].

Beyond molecular design, LLMs are beginning to reshape how clinicians interact with complex infectious disease data [4,5,6]. General-purpose LLMs like GPT-4 and domain-specific variants such as BioGPT have demonstrated near-physician performance on licensing exam questions [4,5,6]. Yet, these models currently suffer from hallucinations—plausible but incorrect statements—and a lack of transparency and contextual grounding, which can pose serious patient-safety risks if deployed prematurely in specialist consultations [4,5,6]. Complementary work has highlighted the potential of multimodal LLM-based agents, equipped with vision and audio processing, to assist in real-time outbreak analytics [4,5,6]. For example, advanced vision models can interpret skin or mucosal lesion images in COVID-19, while voice-based ML algorithms detect subtle vocal changes associated with respiratory infections, offering scalable screening tools that could outperform traditional testing in low-resource settings [1,2,3,4,5,6,7].

Imaging plays a pivotal role in diagnosing lower respiratory tract infections across influenza and COVID-19, and deep learning approaches are automating what was once a labor-intensive task. By pretraining on large adult chest X-ray repositories and fine-tuning on pediatric datasets, CNNs now classify World Health Organization–defined radiological pneumonia by outperforming human readers on ambiguous cases [1,2,3,4,5,6,7,8]. This standardized, high-throughput approach to chest X-ray interpretation can accelerate endpoint assessment in vaccine trials and epidemiological studies for influenza and SARS-CoV-2 alike, while reducing inter-observer variability and resource burdens [9,10,11,12,13].

Together, these AI-driven tools—spanning molecular discovery, clinical decision support, immunological prediction, and imaging automation—are coalescing into a multifaceted arsenal against respiratory pandemics. In the present review, we provide an overview of both established and emerging approaches—from structure-based drug and vaccine design to automated radiographic and clinical decision support—and quantify their real-world healthcare benefits, including improvements in diagnostic accuracy, time-to-treatment, vaccine efficacy prediction, and patient outcomes. We examined a spectrum of AI approaches, from machine-learning models (e.g., random forests) to deep neural networks (convolutional and recurrent architectures) and transformer-based language models, which are deployed across influenza, RSV, HIV, and SARS-CoV-2 research. While this work catalogs AI methods applied to these four viral pathogens, it does not itself perform a quantitative Evidence-Based Medicine (EBM) meta-analysis. Rather, it provides a high-level map of algorithmic approaches and highlights opportunities for future work to align these findings with established EBM frameworks.

## 2. Materials and Methods

We conducted a literature review of peer-reviewed publications indexed in PubMed until May 2025. Our search strategy combined disease-specific terms (*Influenza*, *COVID-19*, *SARS-CoV-2*, *HIV*, *RSV*) with AI-related keywords (*Machine Learning*, *Deep Learning*, *Artificial Intelligence*, *Neural Networks*, *Transformers*).

Out of the 2073 studies initially identified, we filtered studies demonstrating the use of AI to facilitate vaccine development, drug discovery, clinical diagnostics, outbreak forecasting, or immune response modeling. Inclusion criteria required original research studies that applied AI or ML techniques to at least one of the four target viruses—seasonal influenza, RSV, HIV, and SARS-CoV-2—and reported quantitative performance metrics (e.g., accuracy). Eligible studies encompassed human clinical investigations, epidemiological analyses, or in silico molecular applications and were published in English. We excluded reviews, commentaries, and editorials that did not present full datasets or primary results, as well as studies lacking any quantitative evaluation. Animal-only or purely in vitro research without clear translational relevance was omitted, along with investigations that did not employ AI or ML methods. Articles not available in full text, manuscripts in non-English, duplicates, and conference abstracts were also excluded. Titles and abstracts were subsequently screened to assess eligibility, followed by full-text review of selected studies. Data were extracted on disease context, AI methods employed, input data types (e.g., imaging, genomics, clinical records, social signals), key outcomes, and comparative performance with traditional approaches. Selected studies were organized by primary application domain: diagnostic support, vaccine response prediction, outbreak modeling, or therapeutic targeting.

**Figure 1:**
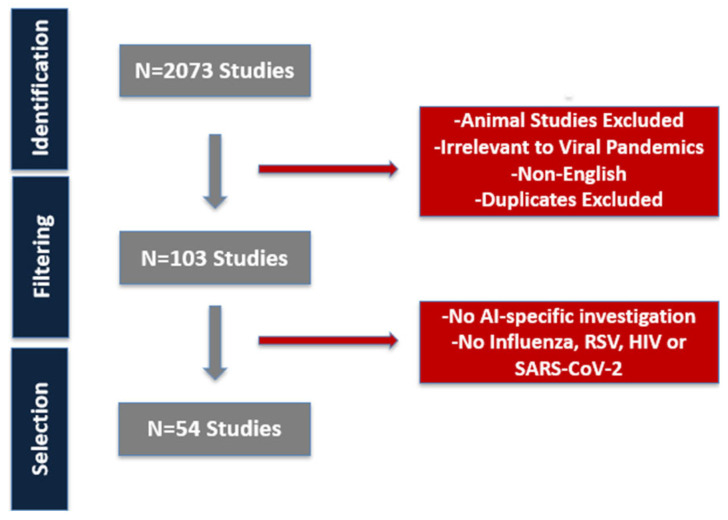
Workflow of literature selection and AI-model categorization. Studies were grouped by viral pathogen and by AI technique.

AI models explored in the included studies ranged from classical machine learning algorithms (e.g., random forests) to more recent platforms such as transformer-based LLM (e.g., GPT-4o, CT-BERT), graph convolutional neural networks, and hybrid epidemic forecasting systems. The final synthesis included representative studies meeting all inclusion criteria (Figure 1). Each was independently reviewed by three researchers, with disagreements resolved by consensus.

## 3. Results

We reviewed 53 AI-driven studies spanning four major pathogens—eight on seasonal influenza, nine on COVID-19, three on RSV, and thirty-three on HIV (Table 1). These works cover diagnostic and prognostic prediction; outbreak and epidemiologic forecasting; genotype–phenotype mapping; vaccine-response modeling; risk stratification; and therapeutic-candidate discovery. They draw on a broad algorithmic toolkit—from optimized back-propagation neural networks and ensemble stacking to Random Forests, support-vector machines, XGBoost, AdaBoost, gradient-boosted trees, convolutional neural networks, transformer-based language models, neural networks, and variational autoencoders. Data inputs include imaging and electronic health records; high-throughput immunoprofiling and large-scale viral sequencing; and real-time social media streams and population-level surveillance statistics—underscoring AI’s scalability and versatility across heterogeneous modalities.

## 4. Discussion

Over the past decade, AI approaches in epidemiology have moved from relatively simple pattern-recognition tools to highly specialized, data-fused systems that can forecast, diagnose, and even anticipate public-health threats in real time. In the 2018–2019 influenza season, for example, early adopters demonstrated that a back-propagation neural network (BPNN) could outperform autoregressive models in short-term ILI forecasting, while others showed that combining decision trees with ensemble stacking yielded a 12 percent reduction in mean absolute error over standard regression baselines [7,8,9]. By mid-2020, CNNs were being used to detect COVID-19 signatures in chest CT scans, and recurrent-CNN hybrids began classifying X-ray images with similar success rates [1,10,11,12,13,14,15,16,17,18]. In late 2021 and over 2022, transformer-based language models such as CT-BERT sifted through clinical reports and social media chatter for early outbreak signals, achieving F1 scores over 0.85 on symptom-extraction tasks [7,17]. Parallel work in HIV monitoring leveraged Long Short-Term Memory networks to predict individual viral-load trajectories from routine lab and demographic data, reducing prediction error by 15 percent versus linear mixed models [59]. More recently, adaptive frameworks like SIMON have automated optimal algorithm selection per region and data type, while multi-model ensembles—integrating compartmental SEIR models with Prophet for trend correction—have set new benchmarks in forecasting accuracy, especially when accounting for emerging RSV seasonality shifts and shifting mobility patterns (Table 1).

**Figure 2:**
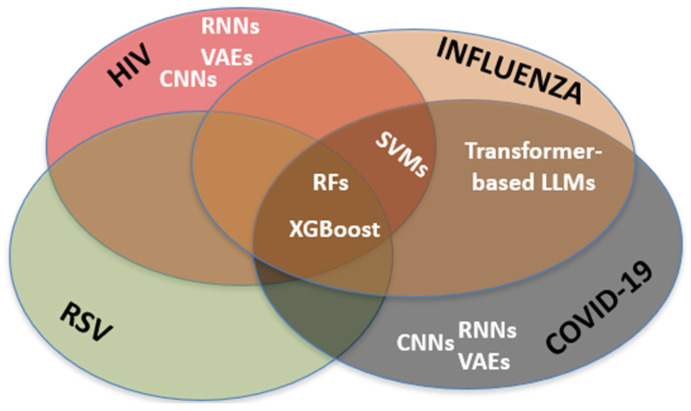
Frequency distribution of AI methods (e.g., Random Forest, XGBoost, CNNs, LLMs) applied across studies targeting influenza, COVID-19, RSV, and HIV [1,7,8,9,10,11,12,13,14,15,16,17,18,19,20,21,22,23,24,25,26,27,28,29,30,31,32,33,34,35,36,37,38,39,40,41,42,43,44,45,46,47,48,49,50,51,52,53,54,55,56,57,58]. This descriptive illustration does not imply endorsement of specific algorithm–disease pairings.

Techniques such as XGBoost paired with Shapley value explanations enable public-health officials to drill down into which features—be they hospitalization counts or Twitter sentiment—drive each prediction [7,13] Equally important has been the fusion of heterogeneous data streams: clinical records and lab results are now routinely enriched with real-world indicators like anonymized mobility data from smartphone apps, hospital-admission dashboards, wastewater surveillance signals—and, in the case of HIV, patient-reported adherence logs and pharmacy refill data (Table 1). This multi-modal integration has bolstered model generalizability, demonstrating that even in an age of deep learning, classic algorithms remain valuable workhorses in the public health AI toolkit (Table 1). Nevertheless, recent successes by transformer-based NLP models and hybrid epidemic forecasters suggest a paradigm shift: adaptability and domain-specific pretraining are becoming hallmarks of next-generation AI in health surveillance. Due to the increasing complexity of methodologies applied across viral infections, we hereby summarize the overlap of AI tools utilized for different pandemics (Figure 2).

**Figure 3:**
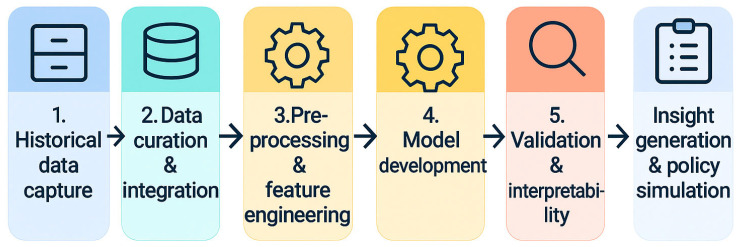
Conceptual AI workflow (data acquisition, preprocessing, feature representation, model training, evaluation, and deployment). This schematic is illustrative and general.

Next, we showcase how diverse AI tools catalogued in the preceding section are most powerful when viewed as parts of a seamless, end-to-end workflow (Figure 3). Beginning with the digitization of century-old case notes, the pipeline highlights how data curation, feature engineering, model development, and interpretability naturally feed into scenario testing that can inform future outbreak responses.

Applications of such AI pipelines across specific viral research topics demonstrate a clear methodological convergence where symptom-only studies rely on gradient-boosted decision-tree families (either XGBoost or random-forest–boosting hybrids) because these algorithms cope well with categorical, low-dimensional inputs and train reliably on modest sample sizes typical of clinical checklists (Table 2). Their predictive power, however, varies based on the pathogen and the clinical context.

### 4.1. Emerging Artificial Intelligence Tools in Viral Pandemics: Opportunities, Challenges, and Future Directions

The latest wave of research into AI during viral pandemics emphasizes the increasing role of LLMs, multimodal AI systems, multi-agent LLM frameworks, and big data analytics in responding to emerging threats such as COVID-19 [2,4,5]. These new developments suggest a significant transformation in how outbreaks are diagnosed, monitored, and managed. Novel AI tools are increasingly incorporated within analytics workflows by regulatory agencies.

The CDC has leveraged AI across multiple viral disease applications, notably using natural language processing to scan free-text reports and detect potential COVID-19 vaccine safety signals, while advancing the use of large language models and spatiotemporal modeling for syndromic surveillance of respiratory viruses [60]. From the ECDC perspective, cognitive technologies such as AI and data analytics are recognized as sufficiently mature for surveillance and forecasting of viral diseases—including influenza [61].

One notable advancement has been the incorporation of LLMs, such as OpenAI’s GPT-4o, into clinical and public health environments [62,63,64,65]. The enhanced vision and voice-processing capabilities of GPT-4o could allow it to serve as an auxiliary diagnostic tool—particularly useful for the preliminary analysis in respiratory illnesses like COVID-19 through voice biomarkers [62,63,64,65]. Early work shows that subtle changes in voice caused by infection-related physiological shifts can be detected using AI, providing an accessible, scalable, and non-invasive means of diagnosis [62,63,64,65]. These models could be deployed via mobile devices in resource-limited settings, offering rapid triage and improving equity in access to healthcare tools [62,63,64,65]. Such applications underscore the potential of LLMs in multimodal environments, where they can process and integrate both textual and non-textual data (e.g., medical images or audio) [62,63,64,65]. This capability expands their use from mere text generation to intelligent assistants capable of real-time evaluations in outbreak scenarios [62,63,64,65]. Furthermore, the rise of multi-agent LLM systems—where multiple specialized agents collaborate or evaluate each other’s outputs—presents a promising approach to improving reliability and accuracy [66,67,68,69,70,71,72]. For instance, an LLM-based diagnostic assistant might generate a preliminary assessment, which is then evaluated or critiqued by a secondary agent trained for safety-checking clinical decisions. These architectures introduce redundancy, reduce error propagation, and help mitigate the risk of harmful outputs from a single flawed model.

Another frontier in AI-enhanced pandemic response lies in large-scale data integration. LLMs and their agents are increasingly being trained on vast and heterogeneous datasets, including genomic sequences, clinical notes, radiological images, and public health records [66,67,68,69,70,71,72]. This capacity for unified processing of multimodal data enables real-time outbreak tracking, forecasting variant evolution, and optimizing resource deployment. When coupled with cloud-based infrastructure and federated learning, such systems can be deployed globally without centralizing sensitive data—an essential feature in maintaining privacy while achieving analytical scale [66,67,68,69,70,71,72].

While LLMs and multi-agent AI frameworks have demonstrated transformative potential during rapidly spreading pandemics such as COVID-19, their effectiveness in the context of chronic or less data-rich viral infections such as HIV and RSV presents a more mixed picture [71,72,73,74,75,76]. HIV, a well-characterized and chronic infection, presents challenges where the LLM example does not naturally align [71,72,73,74,75,76]. Unlike COVID-19, which generated massive, real-time textual, genomic, and clinical datasets, HIV’s clinical data are often longitudinal, sensitive, and siloed due to stigma and privacy issues [71,72,73,74,75,76]. For example, GPT-based tools trained on public health literature may summarize current antiretroviral therapy (ART) guidelines effectively, but they often fail to consider nuanced patient factors such as co-infections (e.g., hepatitis B or tuberculosis), adherence barriers, or socio-behavioral determinants that are critical in managing HIV [71,72,73,74,75,76]. Furthermore, as shown by the incorrect regimen generated for cryptococcal meningitis, reliance on LLMs in HIV care without rigorous cross-verification could be clinically dangerous [2,4,5].

Another limitation lies in the insufficient representation of HIV-specific clinical trials. Many HIV treatment and prevention strategies are still context-dependent (e.g., PrEP in marginalized communities, HIV-positive pregnancies, resistance management), making general-purpose LLMs less suitable for generating personalized recommendations [71,72,73,74,75,76]. While multi-agent frameworks could theoretically allow one model to handle guideline summarization and another to apply patient-specific filters, such implementations have yet to be validated in HIV clinical practice.

In contrast, for RSV, which is seasonal and underreported in many countries, AI systems face challenges in both data sparsity and label reliability [71,72,73,74,75,76]. Unlike COVID-19, where millions of PCR-confirmed cases were logged daily, RSV diagnoses often rely on syndromic proxies in pediatric populations, leading to noisy training inputs [71,72,73,74,75,76]. Multimodal LLMs could again theoretically assist in improving syndromic surveillance by integrating clinical notes, cough audio samples, and seasonality models, but such data is often not consistently labeled or centralized across healthcare systems.

However, there are areas where AI tools, including LLMs, have promise in RSV-related settings. Recent efforts in pediatric emergency departments have explored the use of natural language processing to differentiate viral bronchiolitis from bacterial pneumonia based on clinical notes and imaging reports [71,72,73,74,75,76]. With further training, LLMs could help triage patients or flag high-risk infants for early intervention. Moreover, if trained on global RSV hospitalization data, AI could model seasonal outbreak risk to inform public health vaccination strategies, particularly with the recent approval of RSV vaccines [71,72,73,74,75,76].

Nevertheless, the success of such applications hinges on the integration of standardized, structured data and better annotation of RSV outcomes, both of which are lacking in many settings. LLMs perform well when fed large, diverse, high-quality data—but underperform in fragmented or low-resolution environments [2,4,5]. The future value of LLMs in RSV may depend on initiatives that harmonize pediatric EHR systems, include voice and imaging data, and make labeled datasets openly available for safe model training [2,4,5].

Many performance benchmarks do not account for real-world complexities like data leakage or contamination of training sets—factors that can significantly overstate a model’s clinical utility [2,4,5]. These limitations are further highlighted by the dangers of AI hallucinations in a clinical context [2,4,5]. LLMs can generate plausible-sounding but incorrect statements, which is especially risky in clinical contexts. Current mitigation strategies include the use of retrieval-augmented generation to ground outputs in verified sources and integrating rule-based checks or expert-in-the-loop pipelines [2,4,5]. Protocols increasingly mandate that AI recommendations in diagnostics undergo clinician validation before adoption [2,4,5]. In one striking example, GPT-3.5 confidently recommended an incorrect treatment regimen for cryptococcal meningitis, a life-threatening infection [2,4,5]. More alarmingly, GPT-4, intended to serve as a validation layer, failed to identify the error. This underscores the limitations of single-agent models and the urgent need for validation through multi-agent architectures and human oversight [2,4,5].

These challenges point to the urgent need for more robust evaluation and regulatory frameworks governing the use of AI in viral outbreak response. Information Technology (IT) professionals must prioritize transparency, traceability, and explainability—features that are often secondary in commercial LLM development [2,4,5]. Additionally, interdisciplinary cooperation between AI engineers, epidemiologists, clinicians, and ethicists is essential to establish practical guardrails that align innovation with patient safety [2,4,5].

Beyond methodological limitations, AI systems face substantial obstacles to broad deployment—particularly in low-resource or underserved settings [2,4,5]. Digital illiteracy among both healthcare workers and patients can impede adoption and proper use, while inadequate IT infrastructure further constrains real-world rollout [2,4,5]. Many diseases—especially rare or neglected tropical infections—lack the large, high-quality curated datasets needed to train robust models, leading to performance degradation when data are sparse or skewed. Even where training data exist, models often fail to generalize across different demographic groups, geographic regions, or clinical workflows, risking inequitable outcomes [2,4,5]. Finally, the collection, storage, and processing of sensitive personal health information raise serious privacy and security concerns. Breaches or misuse of data can erode public trust and violate regulatory requirements, underscoring the necessity of end-to-end encryption, federated learning, and rigorous governance frameworks [2,4,5].

Despite these concerns, the advantages of AI in augmenting outbreak response are substantial. LLMs can assist not only with diagnostics but also with synthesizing vast, rapidly evolving bodies of research. During the COVID-19 pandemic, for instance, LLMs were used to summarize clinical trial outcomes, identify emerging variants from genomic data, and support policy simulation efforts [77,78,79,80,81,82,83]. Their ability to adapt across languages and contexts further enhances their suitability for global health applications. Multi-agent LLM systems are especially promising in these settings, as they allow complex reasoning tasks—such as verifying statistical claims or simulating policy impacts—to be distributed across agents, each optimized for a different subtask [2,4,5].

AI applications in healthcare must navigate a complex ethical and regulatory landscape to protect patient rights and maintain public trust. First, data privacy and security are paramount, as patient records contain highly sensitive information, and models trained on these data require robust safeguards against unauthorized access and breaches. Second, consent mechanisms must evolve beyond one-time, blanket approvals to support dynamic, granular consent models that allow individuals to specify which data types may be used and for what purposes, as well as to withdraw consent easily. Third, compliance with existing regulations—such as the EU’s General Data Protection Regulation (GDPR) and the U.S. Health Insurance Portability and Accountability Act (HIPAA)—is essential, but AI’s novel data flows and algorithmic decision-making also call for updated frameworks [84,85]. We recommend implementing standardized documentation practices to ensure transparency. Ethically, AI systems should be governed by multidisciplinary oversight bodies—including clinicians, ethicists, patient advocates, and technologists—that can review algorithms for bias, fairness, and equity before deployment.

### 4.2. Limitations

Despite their promise, AI tools bring a distinct risk profile that must be addressed alongside their benefits. First, algorithmic bias remains pervasive. Large reviews of medical-AI pipelines have shown that imbalanced or poorly annotated training sets can translate into systematically worse performance for minoritised groups, thereby entrenching existing healthcare disparities and even reversing triage priorities when models are deployed in the wild [86]. Second, reliability is still fragile. Multi-modal foundation models routinely hallucinate clinically plausible, but factually wrong, statements. These technical uncertainties are coupled with concerns around the impact on the workforce. Surveys of physicians and nurses document fears of deskilling, reduced clinical judgement, and workflow disruption, as decision-making is shifted to an opaque algorithm [87]. Despite strong results in well-resourced environments, many AI models depend on large, high-quality datasets and powerful computing infrastructures that are often unavailable in low-resource settings. In areas with poor internet access and no standardized electronic health records, these limitations can prevent deployment. To overcome such challenges, we propose that models should be trained across decentralized data sources while preserving patient privacy and forming partnerships with regional health agencies to tailor and validate AI tools according to local disease patterns and data availability.

A methodological constraint of this work is the deliberately high-level nature of cross-algorithm comparisons. In computer science practice, such frequency counts offer, at best, a brief map of community preference, but they do not imply that the most frequently cited model class will outperform all alternatives in every outbreak scenario. Prior benchmarks consistently show that a single off-the-shelf tool rarely suits all viral-disease analyses or trend-prediction tasks, because each pathogen is associated with distinct biological drivers, data modalities, label quality, and class-imbalance profiles. Meaningful model selection, therefore, requires deeper, task-specific experimentation, extensive hyper-parameter sweeps, evaluation under realistic prevalence skews, and multi-algorithm ensembles or entirely novel architectures tailored to niche data characteristics. The engineering burden is non-trivial, where training time can range from hours for a small recurrent model on two seasons of influenza incidence, to several months for iterative pre-training of a pipeline in antiviral drug discovery. These realities reinforce that the work we present should be interpreted as a descriptive and not a prescriptive ranking, and that robust real-world deployment demands pathogen-specific algorithm development, rigorous external validation, and, where necessary, the design of new methods rather than reliance on a single tool.

## 5. Conclusions

AI is enhancing the insights collected throughout the viral pandemic lifecycle, transforming what once was a reactive scramble into a more proactive, data-driven endeavour. Modern surveillance systems now fuse social media signals, mobility data, wastewater assays, and electronic health records, enabling detectors powered by hybrid machine-learning frameworks to flag unusual symptom clusters or anomalous transmission patterns days or even weeks before traditional public health reporting catches up. Meanwhile, ensemble forecasting models that layer mechanistic compartmental approaches with machine-learning corrections have cut prediction errors in half compared with older statistical methods, giving authorities precious extra time to pre-position vaccines, antivirals, and hospital resources.

At the same time, in silico pipelines are accelerating drug discovery and vaccine design. On the vaccine front, reverse-vaccinology workflows powered by transformer architectures scan viral genomes for promising epitope regions, generating candidate peptides that can be synthesized and tested far more quickly than through manual antigen selection.

In clinical settings, multimodal AI systems are beginning to deliver on the promise of rapid, non-invasive diagnostics and decision support. Behind the scenes, multi-agent large language model architectures—where separate agents generate, critique, and validate each other’s outputs—are being explored as a safeguard against dangerous hallucinations, embedding redundancy directly into diagnostic workflows.

Yet, these advances must be viewed alongside persistent challenges. Many AI tools continue to reflect biases in their training data, undermining performance for underrepresented or marginalized groups and raising equity concerns. Validation gaps are especially acute in low-resource settings and for diseases with sparse or noisy data—whether chronic infections like HIV, which generate siloed longitudinal records, or underreported syndromic illnesses such as RSV. On top of this, the overconfidence of generative systems can produce dangerously incorrect clinical suggestions unless there is robust human oversight or automated safety-checking agents in place.

Looking ahead, the full potential of AI in pandemic preparedness and response will hinge not just on ever-more sophisticated algorithms, but on the ecosystem that surrounds them. Building inclusive data infrastructures—secure data-sharing consortia—will be crucial to broadening representation without sacrificing privacy. Equally important are regulatory frameworks and industry standards that mandate transparency, interpretability, and rigorous real-world validation, ensuring that each model can be audited and its decisions traced. Finally, sustained collaboration among researchers, epidemiologists, clinicians, ethicists, and policymakers will be needed to incorporate AI into clinical and public health workflows. Future work should develop open-access, cross-pathogen datasets to enable comparative model benchmarking, integrate explainable AI modules to enhance clinician trust, and pilot AI-driven surveillance platforms for novel pathogens, leveraging real-time genomic and epidemiological data. When these pieces come together, AI will not only modernize infectious disease control but also help build the resilient, equitable healthcare systems that future pandemic threats demand.

## Figures and Tables

**Table 1:** Representative AI studies on influenza, COVID-19, RSV, and HIV.

Author (et al.) (Year)	Disease Studied	AI Method Used	Outcome	Benefit over Traditional Approaches
Seasonal Influenza				
Hu H., et al. (2018) [8]	ILI prediction	AT-optimized BPNN, IAT-BPNN	Delivered MAPE 14.74%, outperforming basic BPNN (MAPE 23.97%) and AT-BPNN (MAPE 20.34%)	Real-time ILI monitoring with near-zero lag; 50–80% error reduction compared to standard BPNN or AT-BPNN; combines social media and official data
Reich NG., et al. (2019) [9]	Seasonal influenza outbreaks	Multi-model ensemble approach using stacking (weighted averaging of 21 predictive models)	Highest forecast accuracy during 2017/18 flu season; superior to all individual and CDC baseline ensemble models	Improved forecast accuracy, reduced variability; integrated into CDC public health responses
Tomic A., et al. (2019) [7]	Influenza vaccine immune response	SIMON automated ML system (Random Forest, discriminant analysis)	Identified novel T-cell subsets predictive of robust vaccine response (AUROC up to 0.86)	Automated high-performing model selection; discovered novel immune signatures
Borkenhagen LK, et al. (2021) [10]	Influenza A virus genotype→phenotype mapping	Random Forest, SVM, neural networks, decision trees, naïve Bayes, k-NN, AdaBoost, logistic regression, Rotation Forest, gradient-boosted trees, hierarchical clustering	RF & SVM consistently highest accuracies; feature-consensus identified critical residue sites	Actionable roadmap for practitioners; feature-consensus map prioritizes experimental follow-up; best-practice checklist enhances model reliability and validation
Wolk DM, et al. (2022) [13]	Influenza complications in vaccine-hesitant populations	XGBoost, Shapley values	Developed GFlu-CxFlag model; outperformed traditional models	Improved risk prediction, targeted interventions
Marquez E., et al. (2023) [14]	Seasonal influenza diagnosis	Supervised ML (Random Forest, bagging, etc.)	RF: Accuracy 0.86	Effective in limited-resource settings; reduced unnecessary molecular tests
Zeng, et al. (2025) [15]	Influenza	LLM for automated symptom-variable extraction; XGBoost and SHAP interpretability	Prediction model: AUC 0.734	The data-driven ILI definition significantly outperformed existing WHO, China CDC, and USA CDC definitions
			New ILI definition (fever ≥ 37.9 °C + cough/rhinorrhea): AUC 0.618	
Hung, et al. (2023) [16]	Influenza	XGBoost	AUC 0.82	Significantly outperforms prior clinical prediction models
COVID-19				
Tian Y., et al. (2023) [17]	Influenza and COVID-19 detection using social media (Saskatchewan, Canada)	Pretrained transformer-based language models: BERT, BERTweet	CT-BERT had best COVID-19 tweet detection (accuracy = 94.6%), BERTweet-best for flu (accuracy = 92.4%)	Showed social media data can support high-accuracy real-time digital surveillance; domain-Specific models (BERTweet) outperformed general ones; complements traditional public health surveillance tools
Keshavarzi Arshadi A., et al. (2020) [1]	Drug and vaccine development	DL, VAE, GCNN, RNN, GAN	Identified drugs targeting SARS-CoV-2 proteins, created comprehensive candidate database CoronaDB-AI for training models	Accelerates discovery through automatic feature extraction, generative models for novel candidates, transfers learned knowledge to overcome data scarcity and improve reliability
Arık SÖ., et al. (2021) [11]	COVID-19 epidemiology forecasting	AI-augmented SEIR model (seq-to-seq, quantile regression)	Consistently superior forecasts	Adapted to dynamic policy/behavior; enabled proactive interventions
Li L., et al. (2021) [12]	COVID-19 vs. CAP	3D CNN	COVID-19 AUC: 0.96 CAP AUC: 0.95	Rapid, precise differentiation between COVID-19 and CAP using CT scans; improved early clinical management
Baccega D., et al. (2024) [18]	COVID-19 forecasting precision	Hybrid ML (Prophet)	Accurate forecasts during variant shifts, outbreaks	Better accuracy, interpretability, lower data needs
Baik, et al. (2023) [19]	COVID-19	CNNs for chest X-ray + MLP, XGBoost & RF for EHR data	Early mortality prediction: ensemble AUC 0.8698	Combining imaging and EHR modalities in a single ensemble markedly improved prognostic accuracy over single-modality models, enabling earlier, data-driven resource allocation
Dipaola, et al. (2023) [20]	COVID-19	TensorFlow combining tabular predictors (age, creatinine, platelets) and NLP-processed text (history, exam, radiology) in a single multimodal model	30-day mortality: AUC 0.87	Integrating unstructured text and structured data markedly improved prognostic accuracy
Akter, et al. (2024) [21]	COVID-19	ARIMA for time-series; multilayer NN with increasing hidden layers; SVMs	SVM yielded most accurate mortality-rate forecasting	The SVM approach substantially outperformed classical ARIMA and standard NN models in predictive accuracy
Zakariaee, et al. (2023) [22]	COVID-19	RF on demographics, clinical manifestations, comorbidities & lab results	Accuracy 97.2%	Combining severity scoring with routine patient data and RF delivered near-perfect mortality prediction, surpassing other ML models
RSV				
Kawamoto, et al. (2024) [23]	RSV infection in pediatric outpatients	XGBoost	AUC-ROC 0.811	Enables remote, non-invasive detection; eliminates need for additional antigen testing in ~75% of patients; reduces discomfort and streamlines diagnosis
Soriano-Arandes, et al. (2025) [24]	Pediatric acute respiratory infections (SARS-CoV-2, RSV, influenza A/B, rhinovirus)	Random Forest and boosting models; SHAP value analysis	RSV AUC 0.81; influenza A/B AUC 0.70; SARS-CoV-2 AUC 0.71	Facilitates early, point-of-care triage based solely on symptoms; reduces reliance on confirmatory laboratory tests; supports rapid, cost-effective decision-making in primary care settings; optimizes resource allocation
Tso, et al. (2022) [25]	RSV in pediatric inpatients	XGBoost on routinely collected EHR data	AUROC 0.919	Provides rapid, non-invasive prediction of RSV positivity using only standard admission data—reducing the need for immediate diagnostic tests and enabling faster infection control measures
HIV				
Mei & Zhao (2018) [26]	HIV-1 and HIV-2 protein classification	SVM; logistic regression; multilayer perceptron	All three models demonstrated superior performance in classifying HIV-1 and HIV-2 proteins	Provided flexible, nonlinear classification, outperforming conventional statistical models
Powell & Davis (2024) [27]	Structural heterogeneity of HIV capsid complexes	Low-dimensional continuous representations	Exceptional performance in reconstructing heterogeneous structures	Enabled data-driven reconstruction of structural heterogeneity beyond traditional tomography
Hu, et al. (2019) [28]	HIV-1 integration site prediction	Attention-based deep learning	Improved prediction accuracy over conventional models	Automatically learned genomic and epigenetic context, offering mechanistic insights
Peng & Zhu (2023) [29]	HIV-1 tropism (monocytes vs. T cells)	Machine learning classification on envelope sequences	Identified five key region features distinguishing proviruses	Enhanced understanding of cell-type tropism beyond basic sequence alignment
Chen, et al. (2019) [30]	HIV-1 tropism prediction	XGBoost; hidden Markov model	High-accuracy tropism prediction	Faster, more interpretable prediction than lab-based tropism assays
Roche, et al. (2024) [31]	HIV self-test image interpretation	Computer vision AI	Detected four infections missed by human readers, showing higher sensitivity	Surpassed human interpretation, improving reliability of field diagnostics
Turbé, et al. (2021) [32]	Rapid HIV field test analysis	Deep learning on test images	Sensitivity 97.8%, outperforming human interpretation	Improved detection of faint lines, reducing false negatives
Benitez, et al. (2020) [33]	ART adherence monitoring	Super learner analysis on EAM data	Enhanced prediction of viral load under constrained optimization	More accurate adherence forecasting versus non-differentiated care
Balzer, et al. (2020) [34]	HIV acquisition risk	ML-based risk scoring	Improved sensitivity for identifying high-risk individuals	More effective risk stratification than traditional scoring systems
Bao, et al. (2021) [35]	HIV and STI risk among MSM	ML on demographic & behavioral data	Promising predictive performance for diagnosis	Early triage using non-lab data, reducing testing burden
Wei, et al. (2019) [36]	HIV-1/HCV co-infection	Naive Bayes; SVM	Identified >20 potential multi-target inhibitors, including approved drugs	Virtual multi-target screening accelerates drug discovery compared to sequential assays
Wang, et al. (2023) [37]	HIV-1/HBV co-infection	Graph neural network-based ensemble	Discovered six novel compounds targeting both viruses	Demonstrated accurate co-infection virtual screening beyond single-target approaches
Onywera, et al. (2020) [38]	HIV/HPV co-infection microbiota	LEfSe analysis on microbiota data	Found increased penile microbiota diversity in co-infected men	Uncovered microbial patterns beyond conventional microbiology methods
Namalinzi, et al. (2024) [39]	Cervical cancer risk in women with HIV	Random Forest	Identified key predictors (disease stage, viral load, etc.)	Improved early detection and reduced costs versus standard screening protocols
Arrigoni, et al. (2023) [40]	HIV-1 protease inhibitor discovery	AI-aided virtual screening	Novel ligand discovered outside known inhibitor classes validated by docking	Expanded chemical space faster than traditional high-throughput screening
Leidner, et al. (2019) [41]	HIV-1 protease ligand potency	Gradient boosting with interaction fingerprints	High accuracy in binding affinity prediction; key interaction features identified	Mechanistic insights into potency beyond conventional QSAR
Kutsal, et al. (2024) [42]	HIV candidate compound identification	LSTM; VAE	Accelerated identification of candidate compounds validated by simulations	Cost-effective pipeline compared to manual lead optimization
Pham, et al. (2024) [43]	ART drug–drug interaction prediction	Deep-ARV with undersampling & ensemble learning	Prediction of four DDI severity categories	Early identification of high-risk interactions improves safety screening
Rawi, et al. (2019) [44]	Resistance to broadly neutralizing antibodies	Gradient boosting machines	Accurate prediction for 33 epitope features identified	Streamlined antibody selection and escape monitoring versus in vitro assays
Steiner, et al. (2020) [45]	HIV drug resistance across antiretrovirals	MLP; RNN; CNN	Enhanced resistance prediction for 18 drugs	Combined architectures improve interpretability and accuracy over single-method models
Cai, et al. (2021) [46]	HIV drug resistance linked to specific mutations	Random Forest; SVM with various kernels	Impact of 21 mutated residues on resistance elucidated	Insight into mutation effects beyond traditional comparative sequence analysis
Blassel, et al. (2021) [47]	HIV RT drug resistance landscape	Machine learning on 55,000 RT sequences	Discovered six novel resistance-associated mutations	Revealed mutation interactions
Altamirano-Flores, et al. (2023) [48]	HIV prognosis via protein motifs	MAREV-1 & MAREV-2 undersampling methods	Identified clinically relevant motif mutations	Guided therapeutic strategies beyond standard genetic marker analysis
Li, et al. (2024) [49]	HIV mortality risk prediction	Random Survival Forests; SVM meta-analysis	Demonstrated strong ML potential for long-term mortality risk	Improved clinical performance versus standard risk scores
Semenova, et al. (2024) [50]	Immunologic signatures of HIV DNA reservoir	Machine learning on immunologic data	Correlated immune cell populations with proviral DNA levels	Provided insights into reservoir dynamics beyond standard virology assays
Ogishi & Yotsuyanagi (2018) [51]	HIV-associated neurocognitive disorder	Machine learning on gp120 env gene features	Identified three amino acid positions that have predictive value	Genetic signature identification, surpassing traditional risk factor analysis
Tu, et al. (2021) [52]	HIV peripheral neuropathy prediction	Logistic regression + ML techniques	Key predictors (infection duration, peak viral load, age) identified; improved classification	Enhanced early prediction over conventional regression alone
Paul, et al. (2020) [53]	Neurocognitive performance in perinatal HIV children	Machine learning classification	Identified children at risk for suboptimal outcomes	Early risk detection, enabling targeted interventions
Petersen, et al. (2022) [54]	White matter brain-age gap in persons with HIV	Gaussian process regression on diffusion imaging	+1.5 years brain-age gap per decade with detectable viral load	Quantitative biomarker for accelerated aging versus standard imaging
Zhang, et al. (2018) [55]	HIV prognosis and mortality via DNA methylation	ML on methylation data	Smoking-associated methylation signatures predicting mortality	Linked molecular methylation markers to clinical outcomes beyond standard clinical indicators
McGowan, et al. (2021) [56]	HIV vaccine antigen identification based on HLA diversity and T cell response	Computational ML tools	Key antigens identified for T cell mediation	Informed vaccine design more precisely than traditional epitope mapping methods
Dănăilă & Buiu (2022) [57]	HIV sensitivity to monoclonal antibodies	Deep learning on amino acid sequences	Predicted strain sensitivity to antibodies	Accelerated identification of potent antibody combinations versus wet-lab screening
Montesi, et al. (2024) [58]	SARS-CoV-2 mRNA vaccine response in people living with HIV	Random Forest on clinical/demographic data	Predicted humoral response, indicating need for boosters	Enabled tailored booster strategies rather than one-size-fits-all vaccination protocols
AdaBoost (Adaptive Boosting), ARIMA (AutoRegressive Integrated Moving Average), ARV (Antiretroviral), AT (Artificial Tree algorithm), AUROC (Area Under the Receiver Operating Characteristic curve), BERT (Bidirectional Encoder Representations from Transformers), BERTweet (BERT for Tweets), BPNN (Back-Propagation Neural Network), CNN (Convolutional Neural Network), CT-BERT (COVID-Twitter BERT), DDI (Drug–Drug Interaction), DL (Deep Learning), EAM (Electronic Adherence Monitoring), EHR (Electronic Health Records), GAN (Generative Adversarial Network), GCNN (Graph Convolutional Neural Network), IAT-BPNN (Improved AT-optimized BPNN), ILI (Influenza-Like Illness), k-NN (k-Nearest Neighbors), LSTM (Long Short-Term Memory), LEfSe (Linear Discriminant Analysis Effect Size), MAPE (Mean Absolute Percentage Error), MAREV (Method for Assessing the Relevance of Each Variable), MLP (Multi-Layer Perceptron), NB (Naïve Bayes), NLP (Natural Language Processing), NN (Neural Networks), OneR (One Rule classifier), PART (Partial decision tree rule learner), RF (Random Forest), RNN (Recurrent Neural Network), SEIR (Susceptible–Exposed–Infectious–Recovered epidemiological model), SHAP (SHapley Additive exPlanations), SIMON (SIMON automated ML system), seq-to-seq (Sequence-to-Sequence model), SMOTE-NC (Synthetic Minority Over-sampling Technique for Nominal and Continuous variables), SVM (Support Vector Machine), VAE (Variational Autoencoders), and XGBoost (eXtreme Gradient Boosting).				

Note: Due to variations in cohort characteristics, endpoints, and reporting metrics, this table is intended as a conceptual overview rather than a quantitative benchmark. Algorithm categories follow the broad schema (Supervised vs. Unsupervised vs. Deep Learning) commonly utilized.

**Table 2:** Performance of symptom-only machine-learning triage models across common respiratory viral infections.

Disease/Setting (Population)	ML Model	Test-Set AUROC	Key Notes
RSV—outpatient ≤ 24 mo (Japan, 4174 visits) [23]	XGBoost	0.811 (95% CI 0.784–0.833)	Remote triage from symptom template
RSV—in-hospital ≤5 y (USA, 54,413 encounters) [25]	XGBoost	0.919 (95% CI 0.906–0.932)	Uses vitals + demographics recorded in first 2 h to flag likely positives
RSV—primary care (Catalonia, 868 children) [24]	Random Forest + Boosting	0.81 (sens 0.64; spec 0.77)	Symptom checklist only; SMOTE-NC for class balance
SARS-CoV-2—same cohort [24]	Random Forest + Boosting	0.71	Best features: absence of wheezing ruled out infection
Influenza A/B—same cohort [24]	Random Forest + Boosting	0.7	Lower discrimination due to overlapping symptom profile
Seasonal Influenza—China, ED/fever clinics (200,135 cases) [15]	XGBoost (Boruta-selected symptoms)	0.734 (CI 0.710–0.750)	Retrospective 2022–2023; model also yielded new data-driven ILI definition
Seasonal Influenza—US & Taiwan, multicenter ED ILI cohort [16]	XGBoost	0.82 (CI 0.79–0.85)	Prospective 2015–2020; outperformed three classic clinical rules

## Data Availability

No new data were created or analyzed in this study. Data sharing is not applicable to this article.
